# Women's Health Surveillance Report: Introduction

**DOI:** 10.1186/1472-6874-4-S1-S1

**Published:** 2004-08-25

**Authors:** Marie DesMeules, Arminée Kazanjian, Heather Maclean, Jennifer Payne, Donna E Stewart, Bilkis Vissandjée

**Affiliations:** 1 Centre for Chronic Disease Prevention and Control, Health Canada, 120 Colonnade Rd, Ottawa, Canada; 2 Faculty of Medicine, The University of British Columbia, 5804 Fairview Ave, Vancouver, Canada; 3 Centre for Research in Women's Health, 790 Bay St., 7th Floor, Toronto, Canada; 4 Centre for Chronic Disease Prevention and Control, Health Canada, 130 Colonnade Rd, Ottawa, Canada; 5 University Health Network Women's Health Program, University of Toronto, 657 University Avenue, ML-2-004, Toronto, Canada, M5G 2N2; 6 School of Nursing Sciences, University of Montreal, Montreal, Canada

## Purpose of the Women's Health Surveillance Report

This report on the health of Canadian women is intended to: (i) determine the extent to which currently available data can be used to provide gender-relevant insights into women's health; (ii) provide information to support the development of health policy, public health programs, and interventions aimed at improving the health of Canadian women; and (iii) serve as the basis for further indicator development. The report provides information and descriptive statistics on determinants of health, health status, and health outcomes for Canadian women. To the extent possible, each chapter presents new, gender-relevant information on a health condition or issue identified as important to women's health during national expert and stakeholder consultations in 1999. Where data or appropriate data are lacking, this is documented. Recommendations for change are made at the end of each chapter, accompanied by a discussion of the gaps in and policy implications of the findings.

## Background to the Women's Health Surveillance Report

A number of jurisdictions have recognized the need for information on gender and health [1-4]. In the fall of 2000, a Steering Committee was formed to undertake the task of producing a national report for Canada using a multidimensional approach that would integrate information from a variety of disciplines. Such a report would serve to monitor progress in women's health and health care and to provide the necessary knowledge base to establish effective policies in health promotion and disease prevention and control.

## Health Determinants

It is generally agreed that differences in health status and health outcomes between individuals-and between men and women-are determined by factors beyond biology. Global forces, including cultural, political, and ecological change, have a powerful effect on health. Against this global backdrop, a complex set of factors-such as socio-cultural and transition experiences, education, income, social status, housing, employment, health services, personal health practices, and the physical environment-comes into play. For example, in developed countries, cultural and economic shifts in attitude toward women's participation in the labour force and control over reproductive decisions have led many women to delay childbirth.

## Approach of the Report

The Women's Health Surveillance Report adopts the broad definition of women's health that provided the framework for the discussion on women and health at the Fourth World Conference on Women (the Beijing Conference), held in September 1995:

Women's health involves women's emotional, social, cultural, spiritual and physical well-being and is determined by the social, political and economic context of women's lives as well as by biology. This broad definition recognizes the validity of women's life experiences and women's own beliefs and experiences of health. Every woman should be provided with the opportunity to achieve, sustain and maintain health as defined by that woman herself to her full potential. [5]

Further, this report attempts to take a gender-sensitive approach to health information where possible, taking into account the context of individual's lives (i.e. the social and cultural roles and responsibilities that differentiate women from men and subgroups of women from other subgroups). Its aim in part is to inform future gender-based analyses.

The authors of individual chapters have made use of population data from large Canadian surveys and administrative databases. Data chosen for analysis depended largely on the availability of the databases at the time of chapter development. Although such data sources can provide interesting insights, they also have limitations. For example, while they usually include a breakdown of the data by sex, they often do not provide sufficient measures by which to explore the influence of gender as determined by the context of women's lives. For example, depression is a major cause of disability worldwide. In Canada, as in other developed countries, the prevalence of depression is the same among boys and girls. After puberty, however, women are about twice as likely as men to experience a depressive episode [6,7]. Traditional surveillance, such as hospitalization data or physician visits for depression, provides the data on these sex differences. What it does not provide is an analysis of how depression in women varies with income, ethnic background, education, and work experience, or how women's roles can shape their susceptibility to this condition (e.g. working double-duty shifts at home and in paid work while possibly experiencing harassment or abuse in either setting).*

## Note

* The views expressed in this report do not necessarily represent the views of the Canadian Population Health Initiative, the Canadian Institute for Health Information or Health Canada.

## References

[B1] Advisory Committee on Women's Health Surveillance (1999). Women's health surveillance: A plan of action for health Canada. Ottawa: Health Canada.

[B2] Women's Health Bureau (2000). Provincial profile of women's health: a statistical overview of health indicators for women in British Columbia. Ottawa: Health Canada.

[B3] Stewart DE, Cheung AM, Ferris LE, Hyman I, Cohen MM, Williams JI (2002). Ontario Women's Health Status Report. Prepared for the Ontario Women's Health Council by The University Health Network Women's Health Program, The Centre for Research in Women's Health and The Institute for Clinical Evaluative Sciences.

[B4] Colman R (2000). Women's health in Atlantic Canada: a statistical portrait. Halifax: Maritime Centre of Excellence for Women's Health. Atlantic Region Fora on Women's Health and Wellbeing.

[B5] (2000). National Women's Law Centre, FOCUS on Health & Leadership for Women, Center for Clinical Epidemiology and Biostatistics, UoPSoM, the Lewin Group. Making the grade on women's health: a national and state-by-state report card.

[B6] Phillips S (1995). The social context of women's health: goals and objectives for medical education. Can Med Assoc J.

[B7] Stewart DE, Rondon M, Damiani G, Honikman J (2001). International psychosocial and systemic issues in women's mental health. Arch Women's Mental Health.

